# COVID-19 and MAFLD/NAFLD: An updated review

**DOI:** 10.3389/fmed.2023.1126491

**Published:** 2023-03-24

**Authors:** Ali Nowroozi, Sara Momtazmanesh, Nima Rezaei

**Affiliations:** ^1^School of Medicine, Tehran University of Medical Sciences, Tehran, Iran; ^2^Network of Immunity in Infection, Malignancy and Autoimmunity (NIIMA), Universal Scientific Education and Research Network (USERN), Tehran, Iran; ^3^Research Center for Immunodeficiencies, Pediatrics Center of Excellence, Children's Medical Center, Tehran University of Medical Sciences, Tehran, Iran; ^4^Department of Immunology, School of Medicine, Tehran University of Medical Sciences, Tehran, Iran

**Keywords:** COVID-19, MAFLD (metabolic associated fatty liver disease), NAFLD (non alcoholic fatty liver disease), NASH (non-alcoholic steatohepatitis), metabolic syndome, vaccine, SARS-CoV-2

## Abstract

The COVID-19 pandemic is ongoing and places a substantial burden on healthcare systems worldwide. As we further shed light on different disease characteristics, we identify more and more groups of people at higher risk of poor COVID-19 outcomes. Metabolic-associated fatty liver disease (MAFLD) (previously non-alcoholic fatty liver disease or NAFLD) is a common metabolic disorder characterized by fat accumulation and liver fibrosis. Given its close correlation with metabolic syndrome, an established risk factor for severe COVID-19, it is necessary to investigate its interplay with the novel coronavirus. In this study, we review the available data on COVID-19 prognosis, treatment and prevention options in patients with MAFLD, and the effect that the disease and the pandemic have on MAFLD care. Furthermore, we point out the gaps in the current literature to accentuate the work that needs to be done to improve MAFLD care during the pandemic and beyond.

## 1. Introduction

The coronavirus disease 2019 (COVID-19) pandemic caused by severe acute respiratory syndrome coronavirus 2 (SARS-CoV-2) has posed a daunting challenge since late 2019, with approximately 600 million confirmed cases and 7 million deaths as of September 1st, 2022 (1). Early on, we learned that while respiratory symptoms may be predominant in COVID-19, the disease affects various organ systems, with gastrointestinal, cardiovascular, neurological, hematological, and renal involvement (2–10). With the growing knowledge of the disease, we learned that in addition to the acute phase, COVID-19 might induce multi-system long-term consequences (i.e., long COVID), such as fatigue, myalgia, psychological symptoms, and hepatitis (11–13).

Liver damage is one of the most important aspects of COVID-19, with elevated liver enzymes appearing in approximately 15–65% of patients in the acute phase (14, 15) and prolonged hepatobiliary complications in some cases (16, 17). In critically ill COVID-19 patients, pathological studies have revealed mild lobular and portal inflammation as well as moderate macrovesicular steatosis (18, 19). Direct viral cytotoxic effects, systemic inflammation, hypoxia, coagulopathy, and drug-induced liver injury are all potential causes of liver damage (4). Notably, viral RNA has been detected in liver samples, and SARS-CoV-2 isolated from liver tissue is infectious (20–22). Angiotensin-converting enzyme 2 (ACE2) is the primary viral receptor for SARS-COV-2. The host transmembrane serine protease 2 (TMPRSS2) is also crucial for viral infectiousness. ACE2 and TMPRSS2 were found to be highly expressed in the liver. Cholangiocytes had the highest levels of ACE2 expression, followed by hepatocytes. Transmembrane serine protease 2 was found to be mainly expressed in cholangiocytes, hepatocytes, periportal liver sinusoidal endothelial cells, and erythroid cells. (23, 24). Interestingly, hypoxia and inflammatory conditions were found to upregulate ACE-2 expression (25, 26).

The liver injury could be more severe in patients with pre-existing chronic liver diseases. This can be partly explained by the increased expression of ACE2 in these patients (26–28). Non-alcoholic fatty liver disease (NAFLD), recently known as metabolic-associated fatty liver disease (MAFLD), is a spectrum of diseases ranging from simple steatosis with or without mild inflammation to a necroinflammatory subtype with the presence of hepatocellular injury (non-alcoholic steatohepatitis (NASH)) and cirrhosis (29, 30). NAFLD is the most common cause of chronic liver disease and is estimated to have affected a quarter of the global population (31, 32). Of note, given the role of cardiometabolic risk factors in the development and progression of the disease, two new position papers (29, 30) proposed the terminology of MAFLD instead of NAFLD in 2020 to better capture the pathophysiology of the disease (33, 34).

Though controversial, early reports during the pandemic indicated that patients with NAFLD have a greater risk of developing a more severe disease course (35–37). Given the association of MAFLD with other cardiometabolic risk factors, which are also well-established predictors of poor prognosis in COVID-19, it remains unclear whether MAFLD is merely associated with poor outcomes or plays a causal role. Moreover, not only can MAFLD influence the course of COVID-19, but it is also important to recognize the effects of the COVID-19 pandemic on the care of patients with MAFLD and the epidemiology of the disease. In addition, given the global scale of COVID-19 vaccination, the focus of research should shift to the safety and efficacy of COVID-19 vaccines in patients with MAFLD.

**Figure 1:**
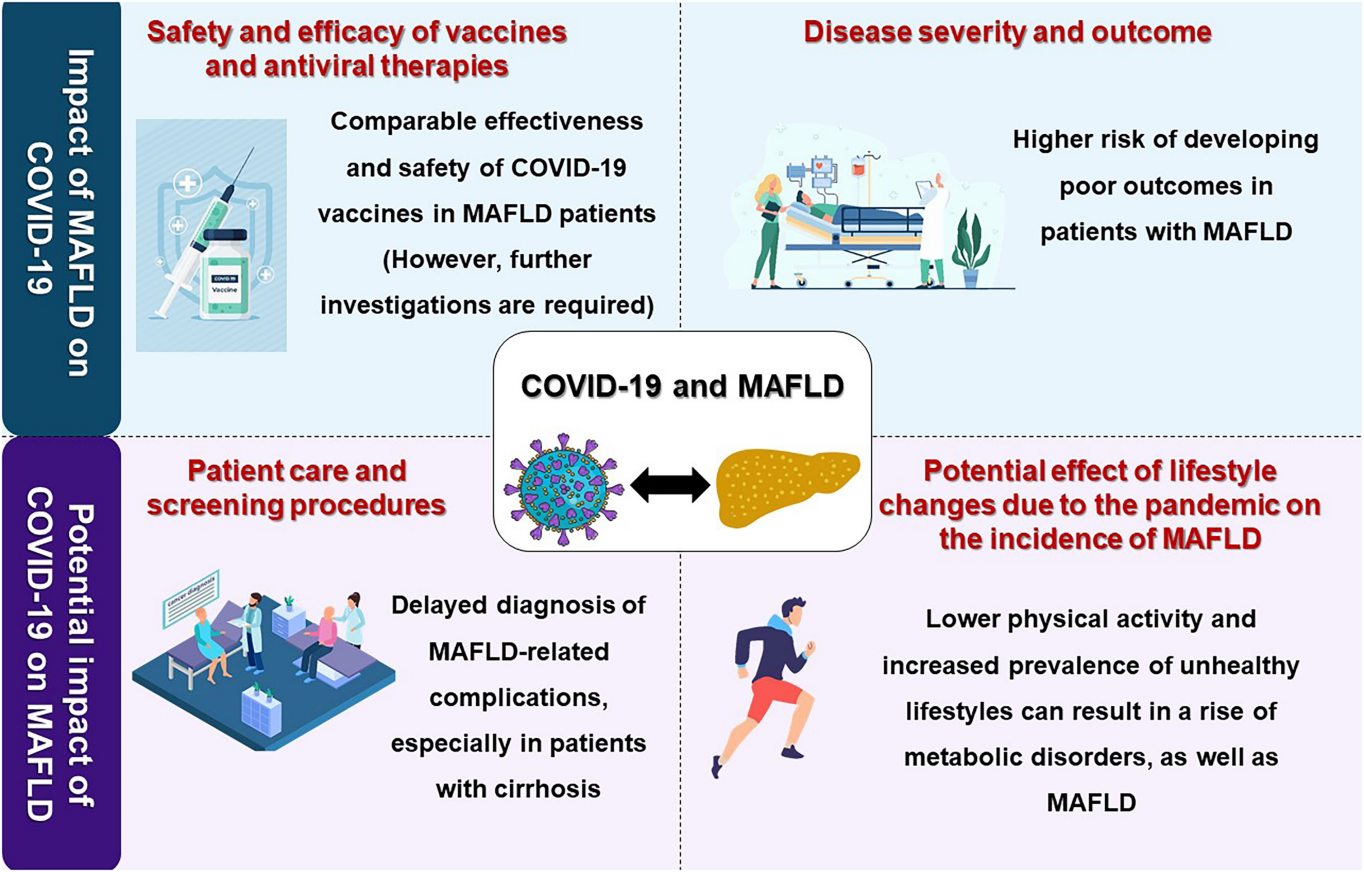
A visual summary of the interplay of the COVID-19 pandemic and metabolic-associated fatty liver disease (MAFLD). Image made using material provided by pch.vector on Freepik.

In this review, we provide a concise yet comprehensive overview of the interplay between MAFLD and the COVID-19 pandemic, focusing on the COVID-19 outcomes in patients with MAFLD, the impact of the COVID-19 pandemic on the care of patients with MAFLD and the epidemiology of the disease, and COVID-19 vaccination in patients with MAFLD (Figure 1). We also pave the way for future research by highlighting the current gaps in the field’s knowledge.

## 2. COVID-19 in patients with MAFLD

Metabolic factors such as obesity and diabetes are established risk factors for severe COVID-19 (38). Hence, it is only logical to assume that MAFLD is associated with a worse prognosis for COVID-19 (39). While almost all studies show that patients with MAFLD are at a higher risk of severe disease, it is not yet well understood whether MAFLD-related changes can act as an independent prognostic factor and, if they do, to what extent they can impact the clinical course of COVID-19. The following sections review the various aspects of the MAFLD-COVID-19 interaction.

### 2.1. COVID-19 severity

According to an analysis of a Korean nationwide cohort, patients with NAFLD had a 35–41% increased disease risk of severe COVID-19 (40), and in another study, patients with MAFLD were four times more likely to acquire severe disease (adjusted odds ratio (OR) = 4.07, *p* = 0.02) (41). A study in Turkey (42) showed that patients with hepatosteatosis (HS) had significantly higher pneumonia severity scores compared with non-HS patients (*p* < 0.001). Among hospitalized SARS-CoV-2 infected patients with NAFLD, diabetes and advanced liver fibrosis were independent predictors of progression to severe disease (adjusted ORs = 8.26 and 11.06 (*p* = 0.03 for both), respectively) (43). Targher et al. (44) reported that patients with intermediate and high Fibrosis-4 (FIB-4) scores had more than four and five times higher risk of severe COVID-19, respectively, compared with patients without MAFLD. Even when adjusted for sex, obesity, and prior diabetes history, the odds of severe disease remained high (OR = 2.59, *p* = 0.03 for intermediate and OR = 4.04, *p* = 0.02 for high FIB-4 scores). Intermediate and high FIB-4 scores had a combined 3-fold increase in severe COVID-19 risk (adjusted OR = 2.95, *p* < 0.005). Similarly, in a study in Italy (45), the FIB-4 score < 1.45 was associated with lower disease severity (adjusted OR = 0.3, *p* = 0.01) and mortality (adjusted OR = 0.4, *p* = 0.04). Contrary to the previous results, do Amaral e Castro and colleagues (46) did not find an association between HS and worse COVID-19 outcomes, although HS was more common among patients with worse outcomes. Furthermore, one study (47) found increased risks of intensive care unit (ICU) admission and mortality with increasing liver fibrosis degree in univariable analysis, although they became insignificant when the risk was adjusted for other factors. A recent meta-analysis of 16 studies by Hayat et al. (48) showed a three-fold increase in severe COVID-19 risk in patients with MAFLD compared with controls. ICU admission was also more incident in patients with MAFLD; however, mortality was similar to the control group. Similar results were achieved in a 2021 analysis of adjusted risks (37), with an adjusted OR of 2.6 (*p* < 0.001) for severe disease, 1.66 (p < 0.001) for ICU admission, and 1.01 (*p* = 0.96) for mortality.

### 2.2. Hospitalization and recovery

Corapli et al. (42) observed that patients with HS were more likely to be admitted (65% vs. 48%, *p* = 0.003), with similar ward (*p* = 0.93) and ICU (*p* = 0.50) stay durations in HS and non-HS groups nonetheless. In a preprint (49), each additional year of having NAFLD/NASH was associated with an 86% increase in the risk of hospitalization (*p* < 0.01). An interesting result of this study was that when patients were adjusted for NAFLD/NASH, obesity decreased the chance of hospitalization by almost 60% (p < 0.01), pointing toward the important role of liver fibrosis in COVID-19 prognosis in obese patients. While using medications in the 3 months leading to the COVID-19 diagnosis did not result in less hospitalization, those who had undergone bariatric surgery were less likely to be admitted (OR = 0.22, *p* < 0.05). Furthermore, patients with NAFLD were much more likely to experience disease progression when hospitalized (OR = 6.4) (50). Additionally, these patients recover 36% slower (*p* < 0.001, based on time to or readiness for discharge) and are more likely to face pulmonary thromboembolism (OR = 2.15, *p* = 0.04) (51).

### 2.3. Indirect effects of fatty liver diseases

In addition to being an independent predictor, MAFLD appears to increase the effect of obesity on the prognosis of COVID-19. While obese patients are 1.5 times more likely to acquire severe disease (52), Zheng and colleagues (53) demonstrated that in patients with MAFLD, obesity was associated with six times higher risk for severe COVID-19. Moreover, prolonged viral shedding might also be present in this population (50).

Secondary sclerosing cholangitis (SSC) is a hepatic complication of COVID-19, with an incidence of 11.8% in patients with severe disease (54) and 2.0% among all hospitalized patients (55). Hartl et al. (55) found that among 10 hospitalized patients with COVID-19 who developed SSC, seven (70%) were because of NAFLD/NASH.

### 2.4. The potential underlying mechanisms for the impact of MAFLD on the prognosis of COVID-19

The reason behind these worse outcomes is a matter of debate, with several possible mechanisms involved. Some propose that MAFLD exacerbates COVID-19’s cytokine storm by increasing the release of pro-inflammatory cytokines from the liver (44, 56). In contrast, others hypothesize that innate immunity diminishes with the liver’s immune cell shift from pro-inflammatory M1 macrophages to regulatory M2 macrophages (57), leading to the deterioration of the patient’s condition (50). A recent study (58) has confirmed both of these findings and demonstrated that patients with MAFLD expressed higher levels of some inflammatory cytokines [such as interleukin-6 (IL-6), which has been shown to play an important role in severe disease and its treatment (59)] and lower levels of interferon-γ (IFN-γ), which is crucial to macrophage activity. Another involved mechanism might be the upregulation of SARS-CoV-2 entry proteins (i.e., ACE2 and TMPRSS2) in obese patients with NASH (28). Furthermore, since fatty liver diseases are closely intertwined with metabolic syndrome, similar detrimental pathophysiological pathways are likely involved (42).

## 3. COVID-19 vaccination in patients with MAFLD

Few studies are available on how MAFLD affects COVID-19 vaccination outcomes.

### 3.1. Adverse reactions

Wang et al. (60) found that 24.9% of patients with MALFD who received the Sinopharm (BBIBP-CorV) vaccine (inactivated virus) showed adverse reactions seven days post-inoculation, which is lower than the vaccine’s phase 3 results (more than 40%) (61). In another study (62), patients receiving either Comirnaty (BNT162b2) or CoronaVac were divided into HS (those with moderate/severe hepatosteatosis) and control groups. Patients in the HS group showed fewer adverse reactions after the first and second doses of CoronaVac. In contrast, Comirnaty resulted in a higher rate of systemic reactions in the HS group after the first dose (58% vs. 39%, *p* = 0.008), especially fatigue (40% vs. 27%, *p* = 0.07), and also a higher rate of joint pain after the second dose (13% vs. 1%, *p* < 0.001).

### 3.2. Immunogenicity and effectiveness

In the study by Wang and colleagues (60) (Sinopharm vaccine), seroconversion was observed in 95.5% of the patients, which is comparable to the nearly 100 percent achieved in the phase 3 trial. Moreover, Cheung et al. (62) observed that on day 56 after the first dose, all cases receiving Comirnaty had achieved seroconversion with similar titer levels (*p* = 0.68). However, the best-responding cases (top 25% of virus microneutralization titer levels) were more prevalent in the control group (*p* = 0.04). All HS patients and all controls, except one, remained seroconverted on day 180 after the first dose, with similar titers (*p* = 1.00 for both). CoronaVac also produced similar seroconversion rates in HS and control groups on day 56 (*p* = 0.13); however, the geometric mean titer was lower in the HS group (*p* = 0.02). Similar to Comirnaty, the best responders were mostly from the control group (*p* = 0.04).

## 4. Effect of the COVID-19 pandemic on the care and incidence of MAFLD

NASH is one of the most common causes of cirrhosis and the second leading indication for a liver transplant. Patients with cirrhosis require prompt diagnosis and treatment of the relevant complications. Considering the annual cumulative hepatocellular carcinoma (HCC) incidence rate of 2.6% for NASH-related cirrhosis, these patients need routine screening for HCC (31). Notably, a multicenter investigation found a significantly decreased number of HCC diagnoses and an increased rate of HCC treatment delay compared to the same period in the previous year during a high prevalence of COVID-19 (63). Moreover, regular screening for esophageal varices, given the high risk of mortality, is also required in patients with NASH-related cirrhosis (64); however, during the COVID-19 pandemic, most screening procedures were delayed, which presumably has led to undiagnosed cases as we are recovering from the pandemic (65). One such example is screening endoscopy, which was recommended to be performed only in urgent circumstances by the pandemic guidelines. This has most likely resulted in missed esophageal varices due to delayed screening (66, 67), especially in earlier periods of the pandemic, though to the best of our knowledge, no studies have reported the corresponding data. The COVID-19 pandemic has also adversely affected transplantation activity and, in turn, affected the care of patients with NASH-related cirrhosis (68).

From another perspective, the COVID-19 pandemic caused considerable behavioral changes due to the restrictions, including lockdown, home confinement, and closure of sports facilities. Several studies have shown decreased physical activity and increased prevalence of unhealthy lifestyles, including increased dietary intake, decreased sleep, and increased smoking during COVID-19 lockdowns (69–71). A longitudinal investigation of NAFLD patients showed that the more active patients had lower physical activity than before during the lockdown, while inactive people had higher physical activity (72). Importantly, cohorts of patients with NAFLD showed that COVID-19 lockdown caused a significant increase in body weight, body mass index (BMI), insulin resistance, cholesterol levels, low-density lipoprotein (LDL) levels, and glucose levels. It also led to a reduction in high-density lipoprotein (HDL) levels alongside with progression of fatty liver (73–75). In addition, population-based analyses using the United States (US) national mortality records revealed that the steady increase in NAFLD mortality prior to the COVID-19 pandemic sped up during the pandemic (76). A recently published cohort study comparing patients before and after a COVID-19 lockdown grouped patients with NAFLD according to the level of physical activity. They found that the fatty liver index (FLI) increased in all groups after the lockdown. However, the elevation in the FLI was higher in the medium physical activity than in the low physical activity group (72). In addition to worsening metabolic risk factors and liver involvement in patients with NAFLD, such lifestyle changes may increase the likelihood of an increased incidence of NAFLD in the coming years as we recover from the pandemic, given the strong association of metabolic risk factors with NAFLD development and progression (32).

The COVID-19 pandemic, although catastrophic, provided and continues to provide many valuable lessons to experts and policymakers of all fields, especially medicine. First and foremost, healthcare providers must always anticipate a sudden crisis that halts delivering health services. Although the COVID-19 pandemic pressured organizations into identifying actions and developing protocols to counter the effect of this global crisis, the effort must be continuous and the results have to be updated regularly based on most recent evidence. Telemedicine is an example of a tool that has been extensively studied during the ongoing pandemic and shown to be effective (77, 78) and satisfactory (79); hence, we suggest healthcare facilities commence and test out different telemedicine approaches to identify the most suitable one for their use. Rapid re-initiation of medical practices during crises is of utmost importance; however, this must not result in the normalization of the ongoing calamity. While continuing care for some patients is necessary, some medical practices could be postponed with no or minimal adverse outcomes (80). Comprehensive crisis-management guidelines and protocols are required to stratify medical services based their delay capacity. Undoubtedly, in the unfortunate event of a similar disaster in the future, the world’s response would be much more appropriate given the experience gained from COVID-19, just as it was for COVID-19 because of the previous outbreaks such as Middle East Respiratory Syndrome (MERS) and SARS (81).

## 5. Limitations and future directions

Current studies, as presented in this manuscript, provide strong evidence that patients with MAFLD tend to experience worse COVID-19 outcomes. However, literature is short of approaches to mitigate this risk. Consequently, to the best of our knowledge, no specific COVID-19 guidelines have been developed for patients with MAFLD. We believe the focus of future related studies should be on evaluating different care strategies in these patients.

Vaccination and specific antiviral treatments are the current trend in COVID-19 research. To the best of our knowledge, very few studies have evaluated COVID-19 vaccine effectiveness in patients with MAFLD and no studies have evaluated antiviral treatments (such as remdesivir and Paxlovid) in this population. Investigating these subjects in patients with MAFLD is of utmost importance, given the crucial role of the liver in drug metabolization.

Delay in health care and shift of resources toward managing COVID-19 is the pandemic’s predominant impact on other diseases. No comprehensive analyses have yet been performed to check whether MAFLD prevalence and incidence have increased in this period. Furthermore, there are no studies investigating whether MAFLD complications such as esophageal varices and HCC have significantly increased, given the cardinal role of screening in their detection and prevention.

## 6. Conclusion

Almost 3 years after the emergence of COVID-19, we very well know that it can affect the liver, and patients with certain comorbidities, such as metabolic dysfunction, are at higher risk for severe disease or mortality. Herein, we concisely reviewed the substantial evidence supporting the mutual association between MAFLD and COVID-19. Patients with MAFLD are at a higher risk of poor outcomes during COVID-19, even after controlling for the confounding effect of the other metabolic abnormalities. COVID-19 caused drastic changes in human lifestyle, screening programs, and transplantation programs, which could adversely affect the care of patients with MAFLD, especially those with NAFLD cirrhosis, or even potentially increase the incidence rate of MAFLD in years to come. Assessment of the efficacy and safety of COVID-19 vaccines in this group of patients has garnered attention recently, with the wide global vaccination showing comparable results with the healthy population. Further investigations are required for development of guidelines for management of MAFLD during the COVID-19 pandemic, and assessment of efficacy of vaccination and antiviral therapies in this group of patients.

## Author contributions

AN: conceptualization, investigation, writing—original draft, and writing—review & editing. SM: conceptualization, investigation, writing—original draft, writing—review & editing, and visualization. NR: conceptualization, writing—review & editing, and supervision. All authors contributed to the article and approved the submitted version.

## Conflict of interest

The authors declare that the research was conducted in the absence of any commercial or financial relationships that could be construed as a potential conflict of interest.

## Publisher’s note

All claims expressed in this article are solely those of the authors and do not necessarily represent those of their affiliated organizations, or those of the publisher, the editors and the reviewers. Any product that may be evaluated in this article, or claim that may be made by its manufacturer, is not guaranteed or endorsed by the publisher.

## Abbreviations

ACE2, angiotensin-converting enzyme 2
BMI, body mass index
COVID-19, coronavirus disease 2019
FIB-4, fibrosis-4
FLI, fatty liver index
HCC, hepatocellular carcinoma
HS, hepatosteatosis
ICU, intensive care unit
IFN-γ, interferon-γ
IL-6, interleukin-6
MAFLD, metabolic associated fatty liver disease
NAFLD, Non-alcoholic fatty liver disease
NASH, non-alcoholic steatohepatitis
OR, odds ratio
SARS-COV-2, severe acute respiratory syndrome coronavirus 2
TMPRSS2, transmembrane serine protease 2
